# Effect of Metal Thickness on the Sensitivity of Crack-Based Sensors

**DOI:** 10.3390/s18092872

**Published:** 2018-08-31

**Authors:** Eunhan Lee, Taewi Kim, Heeseong Suh, Minho Kim, Peter V. Pikhitsa, Seungyong Han, Je-sung Koh, Daeshik Kang

**Affiliations:** 1 Department of Mechanical Engineering, Ajou University, San 5, Woncheon-dong, Yeongtong-gu, Suwon 443-749, Korea; guyehl@ajou.ac.kr (E.L.); rlaxodnl@ajou.ac.kr (T.K.); gmltjd507@ajou.ac.kr (H.S.); hgh7706@ajou.ac.kr (M.K.); 2 Global Frontier Center for Multiscale Energy Systems, Department of Mechanical and Aerospace Engineering, Seoul National University, Seoul 151-742, Korea; peter@snu.ac.kr

**Keywords:** metal thickness, sensitivity, crack density, crack-based sensory system, motion detecting system, flexible sensing system

## Abstract

Among many attempts to make a decent human motion detector in various engineering fields, a mechanical crack-based sensor that deliberately generates and uses nano-scale cracks on a metal deposited thin film is gaining attention for its high sensitivity. While the metal layer of the sensor must be responsible for its high performance, its effects have not received much academic interest. In this paper, we studied the relationship between the thickness of the metal layer and the characteristics of the sensor by depositing a few nanometers of chromium (Cr) and gold (Au) on the PET film. We found that the sensitivity of the crack sensor improves/increases under the following conditions: (1) when Au is thin and Cr is thick; and (2) when the ratio of Au is lower than that of Cr, which also increases the transmittance of the sensor, along with its sensitivity. As we only need a small amount of Au to achieve high sensitivity of the sensor, we have suggested more efficient and economical fabrication methods. With this crack-based sensor, we were able to successfully detect finger motions and to distinguish various signs of American Sign Language (ASL).

## 1. Introduction

In the last few years, many studies on detecting motions using kinematic, multiple accelerations, miniature inertial, magnetic sensors have been reported in various fields [1,2,3,4,5,6,7,8,9]. As these sensors have a limitation in terms of wearability and accuracy, a variety of studies have reported flexible, stretchable and high-sensitivity strain or pressure sensors fabricated by graphene [10,11,12], CNT [13,14,15], and nanowire composite structures [16,17,18]. Among these, Kang et al. [19] have provided an ultrahigh-sensitivity (determined as a gauge factor, GF = ∆*R*/$R_{0}$)/ϵ) of about 2000, defined through the normalized resistance (R/$R_{0}$) variation with the strain (ϵ), multifunctional nanoscale crack-based sensor inspired by a spider’s sensory system. By using this sensor, the pressure of a ladybird, pulse, human speech, and flow rate as a function of time were successfully measured. Since this paper was published, many subsequent studies on topics such as enhancing performance, durability, and transparency, while considering diverse materials, have been carried out [20,21,22,23,24,25,26,27]. Most of the crack sensors studied were fabricated by depositing metals on a polymer substrate. These deposited metals, then, may change the features of these sensors, which makes predicting their characteristics challenging.

In this paper, we study the relationship between the performance and transmittance of the crack-based sensor and its metal thickness. We present the best combination of metal thickness, namely of chromium (Cr) and gold (Au), with respect to the sensitivity and transmittance of the sensor, and the economy and efficiency of the fabrication process. Using the highly sensitive sensor, we made a motion sensing system which can sense and distinguish signs of American Sign Language (ASL). With reference to the results of this study, it is expected that a sensor that fulfils various human needs can be economically and efficiently made. Also, it can be helpful to understand the relationship between the thickness of metal and the performance of sensor.

## 2. Materials and Methods

### 2.1. The Fabrication of a Crack Sensor

A crack-based sensor is composed of three layers. Two metal layers are deposited by a thermal evaporating system (Thermal Evaporation System, DD high tech Co., Gimpo-si, Gyeonggi-do, Korea) on a 6 μm PET film (3026 Mylar thin film, Chemplex, Palm City, FL, USA) substrate. Right before the deposition process, plasma treatment is performed in the oxygen plasma system (CUTE, Femto science Co., Hwaseong-Si, Gyeonggi-do, Korea) at 100 W for 10 min, 0.5 torr pressure, and 30 sccm flow rate. A 5 × 40 mm stainless steel shadow mask is used for constant width and length. Cracks on the metal layers are generated by 2% strain by using a material testing machine (3342 UTM, Instron Co., Norwood, MA, USA).

### 2.2. Resistance Variation Measurement of the Crack Sensor

To measure the sensitivity of the crack-based sensor, tensile testing is processed by using a material testing machine (3342 UTM, Instron Co., Norwood, MA, USA). The sensor is clamped at each end by the grips of the material testing machine, which leaves the intact part of the sensor to be 30 mm in length. The sensor is strained at 500 cycles on 2% strain at 2 mm/min, and its resistance variation measured by using a Labview-based data acquisition system (PXI-4071, National Instruments Inc., Austin, TX, USA). 

## 3. Results

### 3.1. The Crack-Based Sensor

**Figure 1:**
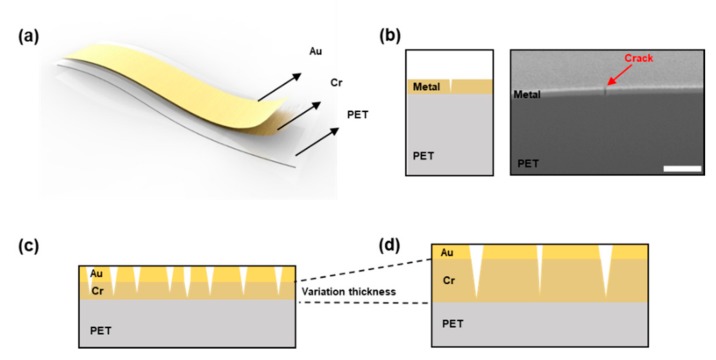
Schematic illustrations and a focused ion beam (FIB) image of the crack sensor. (**a**) Illustration of the crack sensor; (**b**) Cross-sectional FIB image of the crack sensor; Scale bar = 500 nm. (**c**) Illustration of a thin metal layer crack sensor; (**d**) Illustration of a thick metal layer crack sensor.

Figure 1a is the schematic illustration of the crack-based sensor. Two metal layers of Cr and Au are deposited on a PET substrate by a thermal deposition system. Cr is deposited on the PET substrate for crack inducement, and Au is deposited on the Cr layer as an electron conductor. Figure 1b is a cross-sectional scanning electron microscope (SEM) image of the crack-based sensor. A crack that is generated on the metal layers cannot penetrate into the PET substrate in Figure 1b. It can be said, then, that the depth of the crack is same as the thickness of the metal layers. Figure 1c,d shows schematic illustrations of the crack sensory systems for which the depth of the metal layers is different. In Figure 1c, the number of cracks is larger and their depth is shallower than those in Figure 1d. As you can see in Figure 1c,d, the depth of the cracks becomes deeper but their density is lowered when the thickness of the metal layers is thicker.

### 3.2. Resistance Variation of the Crack-Based Sensor and Its Characteristics

**Figure 2:**
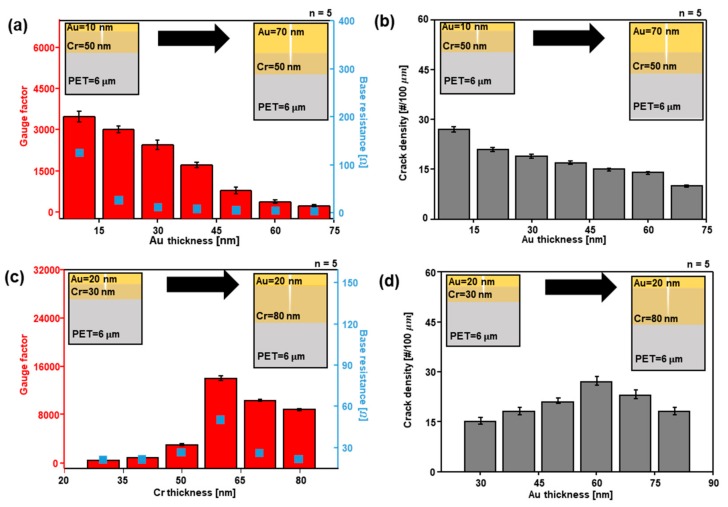
The characteristic variations of the crack sensor according to metal thickness. (**a**) Gauge factor (red column), base resistance (blue squares) changes of the crack sensor according to Au thickness when the Cr thickness is constant at 50 nm; (**b**) Crack density according to Au thickness when the Cr thickness is constant at 50 nm; (**c**) Gauge factor (red column), base resistance (blue squares) changes of the crack sensor according to Cr thickness when the Au thickness is constant at 20 nm; (**d**) Crack density according to Cr thickness when the Au thickness is constant at 20 nm.

Figure 2a presents plots that show the performance of the sensor and base resistance variation versus the thickness of Au from 10 to 70 nm when the Cr thickness is constant at 50 nm. The orange columns are the GF of the crack sensor, and the red squares are its base resistance. When the crack sensor is stretched by an external strain force, the gaps between the nanoscale cracks are changed, leading to a variation in resistance occurring. When the crack gap opens due to external stress, the performance of the sensor improves, in cases where the degree of the maximum resistance is higher than that of the base resistance. In Figure 2a, GF and base resistance are decreased when the Au is thicker. The performance of the sensor decreases, since the reduced degree of maximum resistance is bigger than that of the base resistance. This is due to the crack density effect. The sensitivity of the crack sensor is dominated by the depth and density of the crack [26]. The resistance change of crack sensor occurs due to the variation of the gap between cracks. When the depth or density of the crack becomes deeper, the intervals between cracks change more dramatically when the same amount of strain is applied. Figure 2b is a plot showing that the density of the crack sensor varies according to Au thickness when Cr thickness is constant at 50 nm. When the Au is thicker, the density of the crack sensor decreases. The result of Figure 2b, the reduction of crack density, causes the result of Figure 2a, the decrease of the sensor GF. Thick Cr might have inherent film-stress after deposition, and it can affect the low GF. However, as can be seen in Figure S1, when the inherent film-stress is removed by thermal annealing process, there is no big difference in GF value. Hence, the influence of inherent film-stress can be ignored in this experiment.

Figure 2c is a plot showing the performance of the crack sensor and the base resistance versus the Cr thickness from 30 to 80 nm when the Au thickness is constant at 20 nm. As shown in Figure 2a, the red columns show the GF value of the crack sensor and the blue squares the base resistance value of the crack sensor. In Figure 2c, the GF value of the crack sensor increases until the Cr thickness reaches 60 nm, due to the crack gap opening effect. The greater depth of the cracks causes the crack gap to open wider, resulting in a more dramatic change of the resistance value. When the thickness of Cr becomes greater than 60 nm, the GF value decreases due to the crack density effect. When the thickness of Cr deposited on the PET substrate becomes thicker, the density of the cracks in the metal layer decreases [28,29,30,31]. H. Jin et al. studied the change of the crack density according to the depth of Cr deposited on the PET substrate. They reported that the 15-nm-thick film had denser cracks than those that were 70 and 140 nm thick did. The crack spacings were 2.6, 6.6 and 9.7 μm for the 15-, 70- and 140-nm-thick films, respectively [30]. As can be seen in Figure 2d, the density of the cracks can be seen to vary with the GF value of the crack sensor. If the thickness of the Cr is greater than 70 nm, then, in a case where the tensile is applied to the sensor at 2%, the density of the crack is less influential than the crack opening effect and consequently, the performance of the sensor is decreased. The value of the base resistance also increases until the Cr thickness reaches 60 nm, but decreases when it is thicker than 60 nm.

### 3.3. Performance Variation and Transmittance of the Crack-Based Sensor

**Figure 3:**
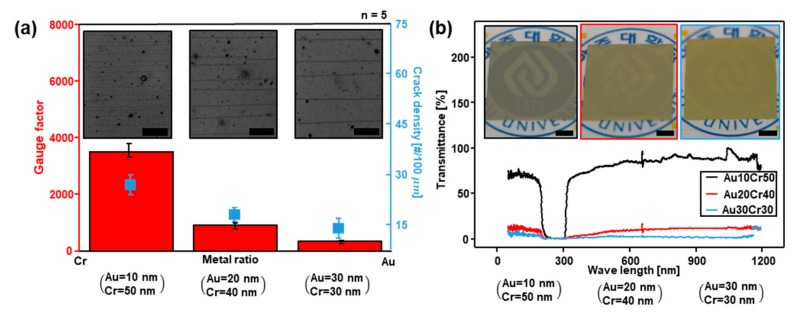
Sensitivity, density and transmittance change of crack sensor according to the ratio of the metal layer. (**a**) Microscopic images and a plot, gauge factor (red column), crack density (blue square) changes according to the metal ratio. Scale bar = 50 μm. (**b**) Transmittance change of crack sensor according to the wave length. Scale bar = 1 cm. (left: Cr50, Au10 Middle: Cr40, Au20, Right: Cr30 Au30).

In the results of Figure 2, we found that the thickness of the metal layer has a great influence on the sensitivity of the sensor. We performed some experiments to find out the relation between sensitivity and metal layer ratio of the crack sensor. Figure 3a is the plot showing the variation of the GF value and the change of the crack density according to the three cases of the ratio of Cr and Au thickness when the metal layer of the crack sensor is constant at 60 nm. The leftmost case is when the thicknesses of Cr and Au are, respectively, 50 nm and 10 nm; the middle case, 40 nm and 20 nm; and the rightmost case, 30 nm for both. The red bars in Figure 3a are the GF of the crack sensor and the blue squares the base resistance of the sensing system. The photos in Figure 3a are the optical images that represent the variation of the crack density. As can be seen in Figure 3a, the GF and the crack density decrease when the ratio of Au is increased. This is due to the width of the crack and its density. The depth of the crack is constant at 60 nm since the thickness of the metal layers is constant. When the ratio of Cr is higher, a large resistance change occurs due to the role of the Cr layer, which is to resist. Conversely, when the ratio of Au is higher than that of Cr, the value of GF is low, as can be seen in Figure 3a due to the role of the Au layer as an electrode. Cr is a more brittle metal than Au. As a result, the density of crack increases when the ratio of Cr is higher than that of Au. As is shown in Figure 3a, the density of cracks should affect to resistance variance by applied strain. Theoretical analysis of the resistance variation according to strain is in supplementary information.

Transparent devices have strong adaptability in various fields such as display panels [32,33,34]. The crack sensor presented in this paper is expected to be used for display panels due to its flexible and sensitive characteristics. Figure 3b is a plot representing the value of transmittance of the crack sensor according to the metal ratio in three cases as in Figure 3a. The grey bars are the value of the transmittance, which is highest when the ratio of Au is low. The photos in Figure 3b are the optical images that represent the difference in crack sensor transmittance, showing the same tendency as in Figure 3a.

### 3.4. Motion Detecting System

**Figure 4:**
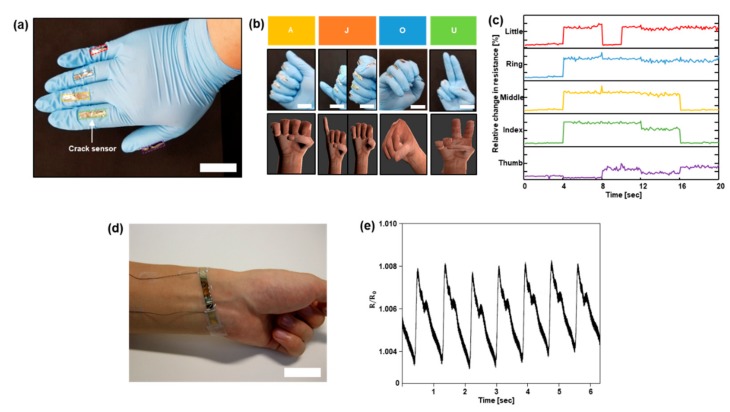
Detecting finger motions and pulse by using the wearable motion detecting system. (**a**) An image of wearable motion detecting system; (**b**) Images and illustrations of four ASL signs. (A, J, O, U); (**c**) Plots representing signals from each finger. (red: little, blue: ring, green: middle, yellow: ring, pink: thumb) Scale bar = 3 cm; (**d**) Images of pulse signal sensing system. Scale bar = 3 cm; (**e**) A plot representing the pulse signal according to time.

Detecting finger motion was conducted based on other technologies, such as wavelet transform of surface EMG signal, integrated with triboelectric nanogenerator and filtering image frame [35,36,37]. We designed a finger motion detecting system using our crack-based sensor. Figure 4a is an image representing a wearable finger motion detecting system. Five crack sensors are attached to each finger of a nitrile examination glove. Using this system, we detected the finger motion and demonstrated the motion in a simulation. Each sensor located in the five fingers, which were connected via a multi-channel data acquisition system, detected the resistance variation. When bending stress was applied to the crack-based sensor, it worked well without being damaged until the angle of the arc was 40° and the radius of curvature ρ was 20 mm (Figure S2). The images in Figure 4b are four ASL signs, A, J, O, and U. Each sensor mounted on five fingers was connected with a microcontroller (Arduino UNO, Ivrea, Italy). The data detected by sensors were transferred to a simulation program (Blender v.2.79), and a virtual hand in the program moves simultaneously with the gloved human hand. As can be seen in the illustrations of Figure 4b, the virtual hand in the Blender can demonstrate the finger motion. Figure 4c shows the plots that present the signals of each finger. The x-axis represents time and the y-axis represents the relative change in resistance from sensors attached to five fingers. The red line is the signal that senses the change of the little finger, the blue line the signal from the ring finger, the green line the middle finger, the yellow line the index finger and the pink line the thumb. The value of resistance changes according to the degree of finger bending. As can be seen in Figure 4a–c, by using the wearable finger motion detecting system, we were able to distinguish and demonstrate some of the ASL signs. Figure 4d is an image showing the pulse sensing system, and Figure 4e is a plot showing the pulse signal according to time.

## 4. Discussion

In this paper, we have reported the variation of the crack sensor sensitivity and transmittance according to its metal thickness. We made the crack sensor by depositing a few nanometers of Cr and Au on 6 μm PET film and conducting 500 cycles of tensile testing from 0 to 2%. When the Au is thin or the Cr is thick, the performance of the crack sensor was noticeable. However, when the Au is thinner than 10 nm, the layer is not able to perform its role as the electrode. Similarly, when the Cr is thicker than 60 nm, its sensitivity becomes lower due to the decrease in the crack density. When the ratio of Au is low, the sensitivity and transmittance of the crack sensor increase. With this sensor, we can detect human finger motions, and distinguish ASL signs.

## Supplementary Materials

The following are available online at http://www.mdpi.com/1424-8220/18/9/2872/s1, Figure S1: The variation of GF and Base resistance according to annealing process. Red column indicates GF and blue square means the base resistance. The metal thickness of sensor is Cr 50 nm, Au 20 nm. Figure S2: The schematic illustration of crack-based sensor. *l* = 15 mm, *w* = 5 mm, *h* = 6 μm.
