# Supplementary material for: Effect of Metal Thickness on the Sensitivity of Crack-Based Sensors
Source: Sensors (Basel). 2018 Aug 31;18(9):2872. doi: 10.3390/s18092872 (PMC6163335; doi:10.3390/s18092872)
Supplement: Supplementary file 1 [file sensors-18-02872-s001.pdf]

Supplementary Information

Effect of Metal Thickness on Sensitivity of Crack Based Sensor

Eunhan Lee [1], Taewi Kim [1], Heeseong Suh [1], Minho Kim [1], Peter V. Pikhitsa [2], Seungyong Han [1,*], Je-sung Koh [1,*], and Daeshik Kang [1,*]

[1] Department of Mechanical Engineering, Ajou University, San 5, Woncheon-dong, Yeongtong-gu, Suwon 443-749, Republic of Korea; guyehl@ajou.ac.kr (E.L); rlaxodnl@ajou.ac.kr (T.K); gmltjd507@ajou.ac.kr (H.S); hgh7706@ajou.ac.kr (M.K);

[2] Global Frontier Center for Multiscale Energy Systems, Department of Mechanical and Aerospace Engineering, Seoul National University, Seoul 151-742, Korea.; peter@snu.ac.kr (P.P);

* Correspondence: dskang@ajou.ac.kr (D.K); sy84han@ajou.ac.kr (S.H); jskoh@ajou.ac.kr (J.s.K); Tel.: +82-31-219-2345 (D.K); +82-31-219-2685 (S.H); +82-31-219-2353 (J.s.K);

Keywords: metal thickness; sensitivity; crack density; crack-based sensory system; motion detecting system; flexible sensing system

Theory Section

The normalized conductance S of the sensor vs strain ε was determined by the probability function $\mathrm{P}(x)$.

$$\mathrm{S} = \int_{\varepsilon}^{\infty} \mathrm{P}(x)\mathrm{d}x, \tag{S1}$$

We found an equation for P(x) with the only "size" parameter – the strain ε_0 that corresponds to the crack gap width $k\varepsilon_0$ being about the grain size $x_0 = k\varepsilon_0$

$$\mathrm{P}(x)\mathrm{d}x = \mathrm{P}(1/x)/x^2\mathrm{d}x, \tag{S2}$$

where $x = \frac{\varepsilon}{\varepsilon_0}$ and k is the proportionality factor to be defined by relating the crack gap width to the strain. k can be different material realization of parallel crack systems and should be obtained from experiment. The general solution satisfying Equation (S2) is

$$\mathrm{P}(x) = \frac{f(\ln(x))}{x}, \tag{S3}$$

where *f(x)* is an even function of its arguments. The well-known *log-normal* distribution function belonging to the class of a so-called skew distribution with long tails

$$\mathrm{P}(\varepsilon) = \exp(-(\ln(\tfrac{\varepsilon}{\varepsilon_0}))^2/\mu^2)/(\varepsilon\mu\sqrt{\pi}), \tag{S4}$$

satisfies Equation (S3) where μ and B are parameters of the pdf. Among solution (S2), it can be expressed as log-logistic pdf.

$$\mathrm{P}(\varepsilon) = \frac{B}{\varepsilon_0}\frac{(\varepsilon/\varepsilon_0)^{B-1}}{(1+(\varepsilon/\varepsilon_0)^B)^2} \tag{S5}$$

With Equation (S1), Equation (S4) gives for resistance $R = 1/\mathrm{S}$ as a function of strain the following:

$$\mathrm{R} = 2/\left(1 - \mathrm{erf}\left(\frac{\ln(\frac{\varepsilon}{\varepsilon_0})}{\mu}\right)\right), \tag{S6}$$

$\mathrm{erf}(x)$ is the error function. Equations (S1) and (S5) leads to Equation (S7).

$$\mathrm{R} = 1 + (\varepsilon/\varepsilon_0)^B, \tag{S7}$$

In the case of multiple cracks, the Equation (S1) should be modified to Equation (S8).

$$S_{multi} = \int_{\varepsilon}^{\infty} P_1(x)dx \int_{\varepsilon}^{\infty} P_2(x)dx \cdots \int_{\varepsilon}^{\infty} P_n(x)dx, \tag{S8}$$

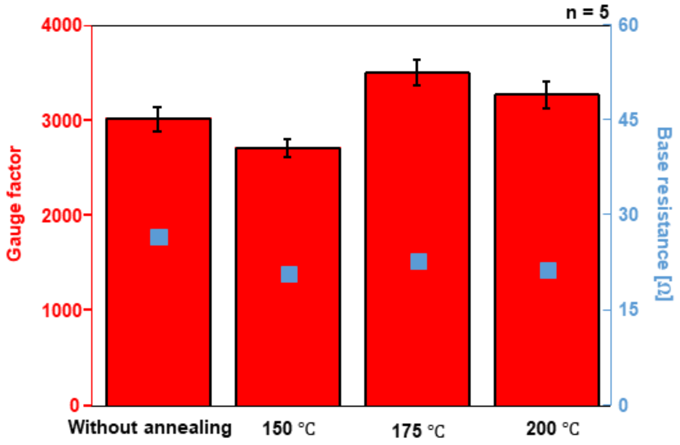

Figure S1. The variation of GF and Base resistance according to annealing process. Red column indicates GF and blue square means the base resistance. The metal thickness of sensor is Cr 50 nm, Au 20 nm.

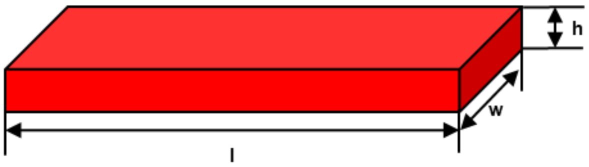

Figure S2. The schematic illustration of crack-based sensor. l = 15 mm, w = 5 mm, h = 6 μm

The thickness of PET is too thicker than that of metal layer so that the thickness of metal layer could be ignored. Because the cross-sectional shape of sensors is rectangular, the neutral plane of sensors y is 3 μm. The crack-based sensor worked until it stretched about 4% strain ε_x. According to the Equation (S9), the sensor worked until the angle of the arc θ is 40° at radius of curvature ρ is 20 mm (Equation S(10)), when the bending stress is applied to it.

$$\varepsilon_x = -\frac{\theta}{l} y, \tag{S9}$$

$$l = \rho\theta. \tag{S10}$$
